# Genome diversity of *Leishmania aethiopica*

**DOI:** 10.3389/fcimb.2023.1147998

**Published:** 2023-04-20

**Authors:** Amber Hadermann, Senne Heeren, Ilse Maes, Jean-Claude Dujardin, Malgorzata Anna Domagalska, Frederik Van den Broeck

**Affiliations:** ^1^ Department of Biomedical Sciences, Institute of Tropical Medicine, Antwerp, Belgium; ^2^ Department of Microbiology, Immunology and Transplantation, Rega Institute for Medical Research, Katholieke Universiteit Leuven, Leuven, Belgium; ^3^ Department of Biomedical Sciences, University of Antwerp, Antwerp, Belgium

**Keywords:** population genomics, hybridization, loss-of-heterozygosity (LOH), asexual evolution, interspecific hybrid, triploid hybrid

## Abstract

*Leishmania aethiopica* is a zoonotic Old World parasite transmitted by Phlebotomine sand flies and causing cutaneous leishmaniasis in Ethiopia and Kenya. Despite a range of clinical manifestations and a high prevalence of treatment failure, *L. aethiopica* is one of the most neglected species of the *Leishmania* genus in terms of scientific attention. Here, we explored the genome diversity of *L. aethiopica* by analyzing the genomes of twenty isolates from Ethiopia. Phylogenomic analyses identified two strains as interspecific hybrids involving *L. aethiopica* as one parent and *L. donovani* and *L. tropica* respectively as the other parent. High levels of genome-wide heterozygosity suggest that these two hybrids are equivalent to F1 progeny that propagated mitotically since the initial hybridization event. Analyses of allelic read depths further revealed that the *L. aethiopica* - *L. tropica* hybrid was diploid and the *L. aethiopica* - *L. donovani* hybrid was triploid, as has been described for other interspecific *Leishmania* hybrids. When focusing on *L. aethiopica*, we show that this species is genetically highly diverse and consists of both asexually evolving strains and groups of recombining parasites. A remarkable observation is that some *L. aethiopica* strains showed an extensive loss of heterozygosity across large regions of the nuclear genome, which likely arose from gene conversion/mitotic recombination. Hence, our prospection of *L. aethiopica* genomics revealed new insights into the genomic consequences of both meiotic and mitotic recombination in *Leishmania*.

## 1 Introduction

*Leishmania* is a vector-borne parasite causing leishmaniasis in humans and animals. Depending on the *Leishmania* species, the disease can present itself in various clinical representations, ranging from cutaneous to visceral leishmaniasis. *Leishmania aethiopica* is an Old World species transmitted by sand flies of the *Phlebotomus* genus, mainly *P. longipes* (Lemma et al., 1969) and *P. pedifer* (Ashford et al., 1973), and is known to cause local (LCL), diffuse (DCL) and the occasional mucocutaneous leishmaniasis (MCL). Where LCL will present as self-healing lesions at the place of inoculation, DCL appears as non-self-healing lesions widespread over the whole body and MCL at the mucosal membranes (i.e. nose, mouth and throat) (Reithinger et al., 2007; Steverding, 2017; Assefa, 2018; van Henten et al., 2018).

*Leishmania aethiopica* is endemic in Ethiopia and the highlands of Kenya, with respectively 1,402 and 398 reported cases of cutaneous leishmaniasis in 2020 (van Henten et al., 2018). The species is considered to be zoonotic and transmitted mainly by *Procavia capensis* and *Heterohyrax brucei* (rock hyraxes) that mostly reside in rocky cliffs (van Henten et al., 2018). Prevalence of active CL ranges from 0.01 to 10.8%, while prevalence including past CL (suspected scar lesions) ranged from 14.0 to 65.4% (van Henten et al., 2018). However, the total burden of *L. aethiopica*-associated disease is difficult to estimate since most of the infections will not result in disease, and if they do, they often remain unreported due to the risk of stigmatization (Reithinger et al., 2007). In addition to being a species that clinically presents three forms of CL, *L. aethiopica* has a high prevalence of treatment failure that remains poorly understood (van Henten et al., 2018). Field isolates of *L. aethiopica* also bear the endosymbiotic and immunogenic double-stranded *Leishmania* RNA virus, which may have potential implications for disease severity (Zangger et al., 2014). Despite these observations of biomedical and epidemiological relevance, research on *L. aethiopica* is almost non-existent, leaving large gaps in the knowledge on the biology of this neglected *Leishmania* species.

Genome diversity studies allow understanding the population dynamics and biology of *Leishmania* parasites, revealing insights into e.g. the epidemic history of the deadly *L. donovani* in the Indian subcontinent (Downing et al., 2011; Imamura et al., 2016), the population structure of *L. panamensis* within its endemic range (Patino et al., 2020; Llanes et al., 2022), the colonization history of *L. infantum* in the Americas (Schwabl et al., 2021), the Pleistocene origin of Andean *Leishmania* parasites (Van den Broeck et al., 2020) and the genetic consequences of hybridization (Rogers et al., 2014; Inbar et al., 2019; Cotton et al., 2020; Glans et al., 2021). Unfortunately, studies on the genetic diversity of *L. aethiopica* are scanty and limited to analyses of microsatellite genotyping, isoenzyme analysis, fragment length polymorphism analyses and/or single gene sequencing (Schönian et al., 2000; Pratlong et al., 2009; Odiwuor et al., 2011a; Krayter et al., 2015). These molecular studies demonstrated that the species *L. aethiopica* is genetically very heterogeneous despite its restricted geographic distribution (Schönian et al., 2000; Pratlong et al., 2009; Krayter et al., 2015) and suggested the existence of a *L. aethiopica/L. donovani* hybrid (MHOM/ET/94/ABAUY) (Odiwuor et al., 2011a). Such findings indicate that the *L. aethiopica*-related disease may be caused by a genetically diverse and recombining species, which may have consequences towards the clinical management and epidemiology of CL in East Africa.

Here, we present the first genome diversity study of *L. aethiopica* to gain a better understanding of the evolutionary history and population biology of this species. We generated a total of 28 *Leishmania* genomes and complemented our dataset with publicly available genomes from *L. donovani*, *L. infantum*, *L. tropica* and *L. major* for comparative purposes. Population genomic and phylogenomic analyses provide genomic confirmation of interspecific hybridization including *L. aethiopica* as one of the two parental species and confirm that *L. aethiopica* is genetically highly diverse. Our prospection of *L. aethiopica* genomics may promote future studies on the genomic basis of treatment failure and clinical outcome.

## 2 Methods

### 2.1 Dataset of genomic sequences

Throughout this manuscript, we use the term ‘strain’ when referring to an isolate that has been characterized (here through whole genome sequencing), ‘clone’ when referring to a strain that has been cloned and ‘genome’ when referring to strains/clones that have been sequenced (Proposals for the nomenclature of salivarian trypanosomes and for the maintenance of reference collections, 1978).

We have sequenced a total of 28 *Leishmania* genomes from Ethiopia within the context of this study (**Supplementary Table 1**), including (i) 20 genomes originating from 19 *L. aethiopica* strains collected between 1972 and 1994, and previously typed as *L. aethiopica* by the Centre National de Référence des Leishmania (Montpellier, France) or the WHO International Leishmania Reference Center (London School of Hygiene and Tropical Medicine, London, United Kingdom). For two strains (GERE and KASSAYE), only the derived clone was sequenced. For one strain (L100), both the strain and a derived clone were sequenced. The L100 strain is the WHO reference strain that represents the same strain as the one used to create the reference genome (https://tritrypdb.org/tritrypdb/app/record/dataset/NCBITAXON_1206056#description). (ii) One genome of the *L. donovani* strain HUSSEN. (iii) One genome of the putative *L. aethiopica/L. tropica* strain L86. (iv) Six genomes of the putative *L. aethiopica/L. donovani* strain ABAUY (hereafter referred to as LEM3469) and its derived clones (LEM3469cl1, LEM3469cl5, LEM3469cl7, LEM3469cl8, LEM3469cl9). All genomes were sequenced (150bp paired-end) on the Illumina sequencing platform of GenomeScan, Leiden, The Netherlands.

For strains L100 and LEM3469, for which we sequenced both the strain and derived clone(s), we here provide information about the number of passages that occurred at ITM before the *in vitro* culturing done within the context of this study. The numbers below reflect the minimum number of passages, as we don’t know how many passages occurred between isolation from patients and shipment from the reference center to ITM. The L100 strain has undergone 29 passages; its derived clone L100cl1 was generated at passage 27 and has undergone a total of 31 passages. Hence, L100 and L100cl1 differ by only 2 passages. The LEM3469 strain has undergone 3 passages; its derived clones were generated after 26 (clones 5, 7 and 8) or 28 (clone 1 and 9) passages, and have undergone a total of 30 (clones 5, 7 and 8) or 34 (clone 1 and 9) passages. Hence, LEM3469 and derived clones differ by about 30 passages, and the clones themselves by either 0 or 4 passages.

For comparative purposes, we also included publicly available sequences from four Old World *Leishmania* species (**Supplementary Table 1**): *L. donovani* from Ethiopia (n=5), *L. infantum* (n=1), *L. major* (n=1) and *L. tropica* (n=1) (González-de la Fuente et al., 2017; Zackay et al., 2018; Warren et al., 2021). We only included one or a few genomes per species with the aim to reveal the parental species of two putative interspecific hybrid strains (LEM3469 and L86) that were included in this study. While we acknowledge that there are many genomes available for the *L. donovani* species complex that would allow us to refine the ancestry analyses of the putative *L. donovani/L. aethiopica* strain LEM3469, a deep phylogenomic study goes beyond the scope of this our study and would not be possible for the putative *L. tropica/L. aethiopica* hybrid strain L86 as there are not many *L. tropica* genomes available.

Altogether, this provided us with a total of 36 genomes for downstream analyses.

### 2.2 Bioinformatic analyses

All sequences were mapped against the *L. aethiopica’s* reference genome L147 (available on tritrypdb.org as TriTrypDB-54_LaethiopicaL14710) using BWA (Li and Durbin, 2010). Resulting SAM-files were converted to BAM-files using SAMtools (Li et al., 2009). All duplicates were marked using the GATK (Genome Analysis ToolKit) software (McKenna et al., 2010). BAM-files were checked for mapping quality by examining flagstat files and coverage was calculated with SAMtools depth to determine the average mapped read depth across the whole genome. Single Nucleotide Polymorphisms (SNPs) and small insertions/deletions (INDELs) were called twice with GATK HaplotypeCaller: once including all 36 *Leishmania* genomes and once including 20 *L. aethiopica* genomes.

SNPs and INDELs were separated with the GATK SelectVariants command. SNPs were filtered based on the GATK hard filter recommendations, including a mapping quality larger than 40 and a quality by depth larger than 2, in combination with a filter that excludes SNPs within SNP clusters (defined by 3 SNPs in windows of 10bp) (Van der Auwera et al., 2013). In addition, SNPs were retained only when the allelic depth per genotype was larger than 5 and the genotype quality was larger than 20 (when genotyping was done across all 35 *Leishmania* genomes) or larger than 40 (when genotyping was done on the 20 *L. aethiopica* genomes). SNPs were annotated using the *L. braziliensis* M2904 annotation file with SNPEFF v4.5 (Cingolani et al., 2012). SNPs were counted per genome for the whole genome and per chromosome in Rstudio (RCoreTeam). Alternate allele read depth frequencies were counted using the vcf2freq.py script (available at github.com/FreBio/mytools/blob/master/vcf2freq.py).

### 2.3 Chromosomal and local copy number variation

Chromosomal and local copy number variations were studied for the 20 *L. aethiopica* genomes and the two interspecific hybrids L86 and LEM3469 based on the per-site read depth as obtained with SAMTools depth. To obtain haploid copy numbers (HCN) for each chromosome, the median chromosomal read depths were divided by the median genome-wide read depth. Somy variation was then obtained by multiplying HCN by two (assuming diploidy for all *L. aethiopica* genomes and L86; see results) or three (assuming trisomy for LEM3469 and its derived clones; see results). To obtain local HCN, the median read depth in non-overlapping 2kb windows was divided by the median genome-wide read depth. These calculations were done using the depth2window.py script (available at github.com/FreBio/mytools/blob/master/depth2window.py).

Windows of reduced or increased HCN were identified by deducting the median HCN across 20 *L. aethiopica* genomes from the HCN as estimated per *Leishmania* genome. This results in a distribution of HCN centered around zero, allowing us to identify 2kb windows with increased (z-score > 5) or decreased (z-score < -5) HCN in each of the 20 *L. aethiopica genomes* and the two interspecific hybrids (LEM3469 and L86). Consecutive 2kb windows showing a significant increase/decrease in HCN were joined by averaging HCN across the consecutive windows, allowing us to detect larger copy number variants. Small deletions/amplifications (i.e. <= 6kb) that do not cover protein coding genes were ignored.

### 2.4 Population genomic and phylogenomic analyses

The quality-filtered SNP VCF files were converted to the fasta format using the vcf2fasta.py script (available at github.com/FreBio/mytools/blob/master/vcf2fasta.py). To get an initial idea on the phylogenetic relationships between the genomes, a phylogenetic network was reconstructed with SplitsTree version 4.17.2 (Huson, 1998; Huson and Bryant, 2006) based on concatenated bi-allelic SNPs. Uncorrected p-distances (proportion of positions at which two sequences differ from each other) were calculated from the SNP genotypes. For the calculated distances, the NeighborNet method (Bryant and Moulton, 2002) was used to generate a split network and the EqualAngle algorithm (Dress and Huson, 2004) was used for final network visualization. For the chromosomal networks, the chromosomal SNPs were selected using BCFtools view and converted into the fasta format for network analyses.

The population structure of *L. aethiopica* was investigated after excluding putative near-identical genomes (see results), i.e. genomes showing fixed differences at <3 sites. In addition, sites exhibiting high linkage disequilibrium (LD) were removed in a pairwise manner (–indep-pairwise) with plink v1.9 (Purcell et al., 2007) within 50 bp windows with a 10 bp step size for three different squared correlation coefficients (r^2 =^ 0.3, 47,244 SNPs retained; r^2 =^ 0.5, 85,725 SNPs retained; r^2 =^ 0.7, 112,241 SNPs retained). ADMIXTURE v1.3 was run for *K* equals 1 to 5 along with a five-fold cross validation [23]. Principal component analysis (PCA) was done using the glPCA function within the Adegenet R-package [25] (RCoreTeam R, 2021). Nucleotide diversity (π), Tajima’s D and Weir and Cockerham’s pairwise F_ST_ (mean and weighted), were calculated in non-overlapping windows of 50kb for the populations as inferred by ADMIXTURE using VCFtools v0.1.13 (Danecek et al., 2011).

Loss-of-Heterozygosity (LOH) regions were identified by analyzing the distribution of heterozygous sites in non-overlapping 10kb windows as described elsewhere (Weir et al., 2016), using the following criteria: min number of SNPs = 1, max number of heterozygous sites allowed per 10kb window = 0, minimum number of contiguous 10kb windows = 4, maximum ⅓ of all 10 kb windows within a LOH region can be gap, max number of heterozygous sites allowed within gap = 2.

The recombination history of *L. aethiopica* was investigated by calculating the LD decay with PopLDdecay v3.41 (Zhang et al., 2019) and the inbreeding coefficient *F*_IS_ after taking into account Wahlund effects. To this end, we considered five strains (117-82, 1561-80, 169-83, 32-83 and 68-83) belonging to the same genetic population as inferred by ADMIXTURE and sampled over a period of four years. *F*_IS_ was calculated as 1-Ho/He, with Ho the observed heterozygosity and He the expected heterozygosity.

## 3 Results

### 3.1 Genomic confirmation of interspecific hybridization including *L. aethiopica* as parent

Sequences were mapped against the *L. aethiopica* L147 reference genome, resulting in a median coverage of 27x to 70x for the publicly available sequence data, and 44x to 119x for the *L. aethiopica* genomes (**Supplementary Table 2**). At least 80% of the positions in the reference genome were covered with at least 20 reads, and 72% of the paired reads aligned in proper pairs (**Supplementary Table 2**). These results show that the coverage of the *L. aethiopica* reference genome was sufficiently large for variant calling, despite the diversity of species included in this study. Genotyping was performed with GATK HaplotypeCaller across all 36 *Leishmania* genomes, revealing a total of 988,363 high-quality bi-allelic SNPs.

**Figure 1:**
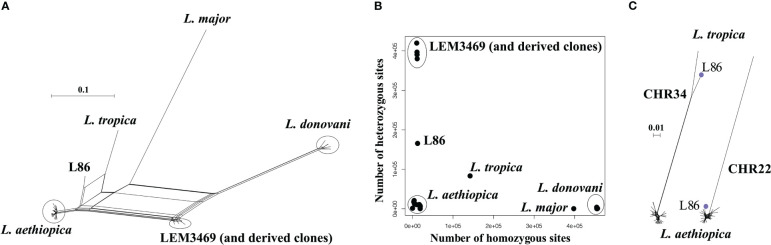
**(A)** Phylogenetic network based on SNPs called across 36 genomes of *L. aethiopica*, the *L. donovani* species complex, *L. major* and *L. tropica*. **(B)** Number of heterozygous sites versus number of homozygous sites for each of the 36 *Leishmania* genomes. **(C)** Phylogenetic network based on SNPs called in the last 700kb of chromosome 34 and the first 300kb in chromosome 22. Blue dot indicates the position of the interspecific *L. aethiopica* - *L. tropica* hybrid L86.

A phylogenetic network revealed a total of four groups of genomes that corresponded to the four species included in this study (**Figure 1A**): *L. aethiopica* (20 genomes), *L. donovani* species complex including *L. donovani* (11 genomes) and *L. infantum* (1 genome), *L. major* (1 genome) and *L. tropica* (1 genome). Two strains did not cluster with any of these species: LEM3469 and its derived clones are positioned in between *L. aethiopica* and the *L. donovani* species complex, and L86 is positioned in between *L. aethiopica* and *L. tropica* (**Figure 1A**). The network shows reticulation and a net-like pattern along the branches of these two strains, suggesting that LEM3469 and L86 are interspecific hybrid parasites or the result of a mixed infection. The observation that the five clones of LEM3469 are also positioned in between *L. aethiopica* and the *L. donovani* species complex indicates that they are hybrids, rather than a mixed infection. Below, we have done additional analyses to confirm that both LEM3469 and L86 are hybrid parasites.

For each genome, we counted the number of homozygous SNPs (where both haplotypes are different from the L147 reference) and heterozygous SNPs (where one haplotype is similar to the L147 reference and the other different). This showed that the *L. donovani* species complex (median 455,265 SNPs), *L. major* (397,822 SNPs) and *L. tropica* (142,059 SNPs) genomes contain a large amount of homozygous SNPs, and are thus genetically distant from *L. aethiopica* L147 (**Figure 1B**). In contrast, L86, LEM3469 (and derived clones) and all *L. aethiopica* genomes contained respectively 12,507, a median 16,941, and a median 11,125 homozygous SNPs (**Figure 1B**). The genomes of uncertain ancestry (L86 and LEM3469) displayed a much larger amount of heterozygous SNPs (165,703 in L87 and a median 390,606 in LEM3469 and derived clones) compared to the *L. aethiopica* genomes (422-20,783 SNPs) (**Figure 1B**). Such high levels of heterozygosity in L87 and LEM3469 further indicate the presence of divergent homologous chromosomes, either as the result of hybridization or because of a mixed infection.

When investigating the distribution of SNPs across the 35 chromosomes in LEM3469 and L86, we found that the majority of chromosomes consists almost entirely of heterozygous SNPs (**Supplementary Table 3**). Exception was one genomic region in L86 (the last 700kb of chromosome 34) that was entirely homozygous for the alternate allele (both haplotypes are thus different from L147). In addition, the first 300kb of chromosome 22 in L86 and chromosomes 9, 10 and 15 in LEM3469 (**Supplementary Table 3**) were virtually devoid of SNPs (both haplotypes are thus similar to L147). This observation of a largely heterozygous genome that is interrupted by homozygous stretches suggests that L86 and LEM3469 are hybrid parasites, rather than the result of a mixed infection.

Similarly to LEM3469, all five derived clones of LEM3469 were SNP-poor in chromosomes 9, 10 and 15 (**Supplementary Figure 1**; **Supplementary Table 3**). All five clones were also SNP-poor in chromosomes 11 and 24, LEM3469 clones 1 and 9 were SNP-poor in chromosome 20, LEM3469 clone 7 was SNP poor in chromosome 1 and LEM3469 clone 8 was SNP poor in chromosome 33 (**Supplementary Figure 1**; **Supplementary Table 3**). Given that LEM3469 clones 1 and 9 were generated after 28 passages and LEM3469 clones 5, 7 and 8 after 26 passages, the loss of SNP diversity in chromosome 20 for clones 1 and 9 must have occurred between passage 26 and 28, while the loss of SNP diversity in chromosome 1 for clone 7 and chromosome 33 for clone 8 must have occurred after they were cloned at passage 26. Our results thus imply a loss of heterozygosity through the process of cloning and culturing of LEM3469.

Phylogenies based on SNPs in genomic regions that were either largely homozygous or SNP-poor in L86 revealed that this strain clustered with either *L. tropica* or *L. aethiopica*, respectively (**Figure 1C**). This clearly points to *L. aethiopica* and *L. tropica* as the two parental species for L86. A phylogeny based on SNP-poor genomic regions showed that LEM3469 and derived clones clustered within the *L. aethiopica* group (see **Supplementary Figure 2** for an example), confirming that *L. aethiopica* is one of the parental species of this hybrid. The absence of largely homozygous genomic regions in LEM3469 and its derived clones precluded us from confirming the other parental species based on phylogenetic analyses alone.

In order to identify and confirm the other parental species of the hybrid parasites, we identified fixed SNPs specific to *L. tropica* (53,477 SNPs), *L. donovani* species complex (297,070 SNPS) and *L. major* (255,443 SNPs). This revealed that 85% of the fixed SNPs specific to *L. tropica* were heterozygous in L86, compared to 0.3% of the *L. donovani* species complex or *L. major*-specific SNPs. Similarly, we found that 91% of the fixed SNPs specific to *L. donovani* species complex were heterozygous in LEM3469, compared to 0.01% of the *L. major* and *L. tropica*-specific SNPs. We next focused on the fixed SNPs that were specific to the six *L. donovani* strains (3,389 SNPs) and the *L. infantum* strain (8,965 SNPs). This revealed that 1% of the *L. infantum*-specific SNPs and 88% of the *L. donovani*-specific SNPs were heterozygous in LEM3469, suggesting *L. donovani* rather than *L. infantum* was the parental species.

Altogether, our results demonstrate that L86 and LEM3469 are the result of hybridization, rather than the result of a mixed infection, between *L. aethiopica* and *L. tropica* (in case of L86) or between *L. aethiopica* and *L. donovani* (in case of LEM3469).

### 3.2 Population genomic structure and diversity of *L. aethiopica*

Variant calling was repeated on a dataset including solely the 20 *L. aethiopica* genomes, i.e. excluding the other Old World *Leishmania* species and the interspecific hybrids LEM3469 and L86. This resulted in a total of 94,581 high-quality INDELs and 284,776 high-quality SNPs called across 20 *L. aethiopica* genomes. Despite the high genome-wide density of SNPs (median 89 SNPs and average 91 SNPs per 10 kb window), we found a low number of heterozygous sites (median 6 SNPs and average 7 SNPs per 10 kb window). The allele frequency spectrum was dominated by low-frequency variants, with 58% of SNPs (170,163 SNPs) occurring at <0.1% of the population. A total of 116,140 SNPs (39.5% of the total) are found within the coding region of the genome, with an approximately equal number of synonymous (56,225 SNPs) and non-synonymous (59,579 SNPs) mutations. A total of 267 mutations had a high impact on protein coding genes, such as the gain of a stop codon (176 SNPs) or the loss of a start codon (41 SNPs). About two third of the high impact mutations (183 SNPs) occurred at low frequency (<= 0.1%) in our panel of 20 genomes. Of the remaining 84 mutations, 66 mutations occurred within hypothetical proteins and only 18 mutations occurred within coding regions with predicted function (**Supplementary Table 4**).

Our panel of *L. aethiopica* genomes displayed substantial differences in terms of the number of SNPs (5,607 - 93,762) and the number of homozygous (243 - 66,068) and heterozygous (2,067 - 69,857) SNP counts (**Table 1**). Most remarkable were i) 1123-81 showing a low number of SNPs (5.607) compared to the other genomes (mean = 82,251; median = 87,132) and ii) L100 and L100cl1 that were almost devoid of heterozygous SNPs (2,071) compared to the other genomes (mean = 32,251; median = 29,985). In addition, the number of fixed SNP differences between genomes ranged between 1 and 76,477 (average = 32,998 SNPs, median = 28,688 SNPs, sd = 20,955 SNPs) (**Supplementary Table 5**). Exceptions were two groups that each contained two near-identical genomes, each pair showing fixed nucleotide differences at only 1 or two sites (**Supplementary Table 5**): i) L100 and L100cl1 and ii) 678-82 and LEM2358cl3, which were sampled nine years from each other and were probably the result of clonal propagation in nature. The sequence similarity (in terms of fixed nucleotide differences) of 678-82 and LEM2358cl3 does not originate from a mix-up because we find the following two differences between both genomes: i) 678-82 is trisomic for chromosomes 30 and 32 while LEM2358cl3 is nearly euploid (see below), and ii) we find single point mutations at 230 sites (i.e. sites where both genomes differ by 1 nucleotide only). These minor differences suggest that both genomes do not originate from cross-contamination. Altogether, our results suggest that the *L. aethiopica* species is highly diverse and consists of both genetically similar and divergent parasites.

**Table 1:** Population genomic statistics for each of the 20 *L. aethiopica* genomes.

Genome	Number of SNPs	Number of HET SNPs	Number of HOM SNPs	Number of LOH regions	Maximum LOH length (KB)	Mean LOH length (KB)	Median LOH length (KB)	Sum of LOH lengths (MB)	Fraction of the nuclear genome covered by LOH
L127	84013	69857	14156	0	NA	NA	NA	NA	NA
LEM3497	88888	34077	54811	8	50	32.5	30	0,26	0,8%
LEM3464	89507	23439	66068	4	140	85.0	85	0,34	1,1%
130-83	68071	35786	32285	12	120	60.8	50	0,73	2,3%
68-83	87450	30164	57286	17	110	45.3	40	0,77	2,5%
169-83	86524	31838	54686	15	130	53.3	40	0,80	2,6%
1464-85	88575	26275	62300	26	70	38.9	30	1,01	3,2%
LEM3498	87132	22275	64857	34	190	57.1	40	1,94	6,2%
32-83	85899	29807	56092	37	180	60.5	40	2,24	7,2%
103-83	93762	35534	58228	43	130	55.6	40	2,39	7,7%
1561-80	88206	25437	62769	40	170	66.8	50	2,67	8,6%
GEREcl7	71473	60956	10517	30	710	115.0	65	3,45	11,1%
678-82	90127	30182	59945	61	640	102.1	70	6,23	20,0%
LEM2358cl3	89660	29485	60175	65	640	96.6	70	6,28	20,2%
117-82	84885	18856	66029	102	270	65.7	50	6,70	21,5%
85-83	92020	27520	64500	79	470	108.9	80	8,60	27,6%
WANDERA	60463	43672	16791	91	710	115.9	80	10,55	33,9%
1123-81	5607	5364	243	147	610	117.1	80	17,22	55,3%
L100cl1	63070	2075	60995	151	1540	168.8	120	25,49	81,9%
L100	63058	2067	60991	154	1530	166.0	120	25,57	82,1%

The genomes have been ordered according to the fraction of the nuclear genome that is covered by a LOH region. SNPs, Single Nucleotide Polymorphisms; HET, heterozygous; HOM, homozygous; LOH, Loss-of-Heterozygosity.

**Figure 2:**
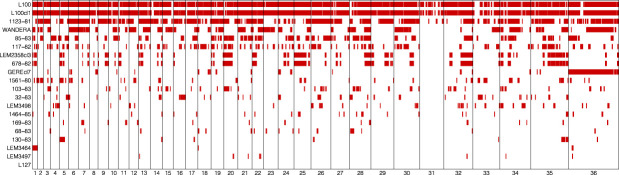
Loss-Of-Heterozygosity regions (red bars) in each of the 20 *L. aethiopica* genomes across the 36 chromosomes.

The large number of fixed SNP differences prompted us to investigate the genome-wide distribution of LOH regions. This effort revealed major differences in the number and length of LOH regions between *L. aethiopica* genomes (**Table 1**) (**Figure 2**). The median length of LOH regions ranged between 30 kb in LEM3497 and 1464-85 to 120 kb in L100 and L100cl1 (**Table 1**). In particular 1123-81, L100 and L100cl1 showed a high number of LOH regions (147 for 1123-81, 151 for L100 and 154 for L100cl1) covering a substantial proportion of their nuclear genome (55.3% for 1123-81, 81.9% for L100 and 82.1% for L100cl1) (**Table 1**) (**Figure 2**). The largest LOH regions were at least 1.5 Mb long (**Table 1**) and were found in chromosome 36 of L100 and L100cl1.

**Figure 3:**
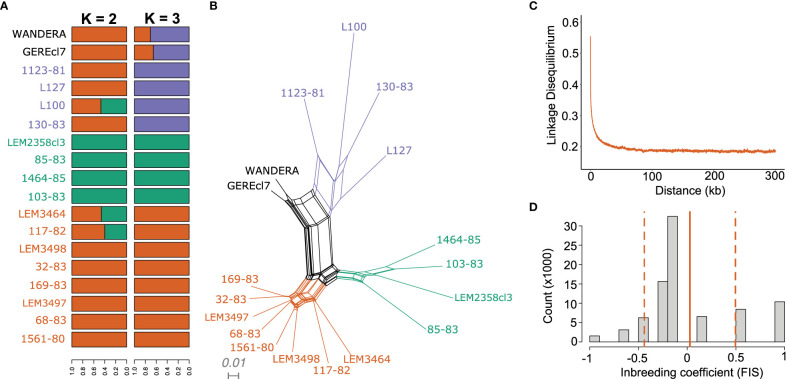
Population genomic diversity and structure of *L. aethiopica*. **(A)** Bar plots depicting ancestral components as inferred by ADMIXTURE for K = 2 and K = 3 populations, based on 85,725 SNPs. **(B)** Phylogenetic network based on uncorrected p-distances among 18 *L. aethiopica* genomes genotyped at 277,156 bi-allelic SNPs. Colored branches and tip labels correspond to the inferred populations by ADMIXTURE at K=3. **(C)** Linkage decay plot for 1561-80, 68-83, 169-83, 32-83 and 117-82 controlling for spatio-temporal Wahlund effects (see methods). **(D)** *F*is distribution after correction for spatio-temporal Wahlund effects for 1561-80, 68-83, 169-83, 32-83 and 117-82. The solid line represents the mean *F*is values (0.027) while the dashed lines represent the standard deviation (± 0.47).

The population genomic diversity and structure of *L. aethiopica* was investigated after removing one genome from the near-identical pairs and excluding sites with high LD. As explained above, we identified two groups of near-identical genomes where each group showed fixed nucleotide differences at only 1 (678-82 and LEM2358cl3) or two (L100 and L100cl1) sites. Here, we randomly excluded 1 genome per group (678-82 and L100cl1) for inference of population structure with ADMIXTURE. This is because the program ADMIXTURE assumes populations are in Hardy-Weinberg Equilibrium and Linkage Equilibrium, an assumption that may be violated when including near-identical genomes that are likely the result of clonal propagation. ADMIXTURE analyses suggested the possible presence of two (K=2) to three (K=3) populations within our panel of *L. aethiopica* genomes, although the 5-fold cross validation was approximately similar for *K*=1 (**Supplementary Figure 3**). The ancestry estimation for the different values of *K* were consistent over the different SNP-pruning thresholds (**Supplementary Figure 2**). Assuming *K*=3 populations, all but two genomes (GEREcl7 and WANDERA) were assigned to one of the three ancestry components with >99% probability (**Figure 3A**). Principal Component Analysis (PCA) (**Supplementary Figure 4**), a phylogenetic network based on genome-wide SNPs (**Figure 3B**) and phylogenetic networks based on SNPs in LOH-poor chromosomes (**Supplementary Figure 4**) showed a clear separation among groups of individuals corresponding to the clusters inferred by ADMIXTURE. These results show congruence in *L. aethiopica* population structure among various inference approaches and suggest that the presence of LOH regions has little impact on the inference process (as shown in **Supplementary Figure 5**). Mean pairwise *F*_ST_ values among these three populations ranged between 0.175 and 0.235, suggesting the presence of population structure (**Supplementary Table 6**).

The phylogenetic network also revealed a reticulated pattern and long terminal branches, indicative of recombination (**Figure 3B**). Estimates of relative recombination rates in *L. aethiopica* were calculated by *F*_IS_ per site and LD decay controlling for spatio-temporal Wahlund effects (see methods) (**Figures 3C, D**). The per-site *F*_IS_ was unimodally distributed and close to zero (mean *F*_IS_ = 0.027 ± 0.47). In addition, LD levels were low with r^2^ descending to 0.2 at 21.9kb. These results suggest frequent genetic exchange in *L. aethiopica*.

### 3.3 Chromosomal and local copy number variations in *L. aethiopica*

The ploidy of all genomes was investigated using the genome-wide distribution of alternate allele read depth frequencies at heterozygous sites (ARDF), which should be centered around 0.5 in diploid organisms. The genome-wide distribution of ARDF was unimodal and centered around 0.5 for all *L. aethiopica* genomes and the *L. aethiopica - L. tropica* hybrid L86, suggesting that the baseline ploidy of these parasites is diploid. However, the distribution of ARDF was bimodal with modes 0.33 and 0.67 for the *L. aethiopica* - *L. donovani* hybrid LEM3469 and its derived clones, suggesting that the baseline ploidy of this hybrid is triploid.

**Figure 4:**
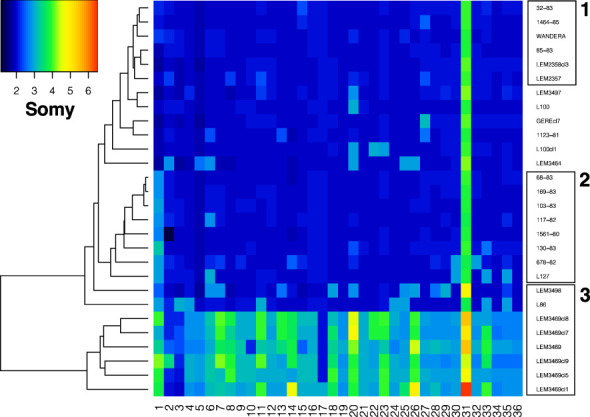
Somy variation across 36 chromosomes for the 28 *Leishmania* genomes sequenced in this study. Box 1 highlights genomes that are nearly diploid, box 2 highlights genomes with a trisomic chromosome 1 and box 3 highlights genomes showing high somy variability (see text).

Variation in chromosomal copy numbers was further investigated using normalized chromosomal read depths (RD). The RD estimates revealed that chromosome 31 was at least tetrasomic in all genomes (**Figure 4**). Little variation was detected for six *L. aethiopica* genomes that were largely diploid (box 1 in **Figure 4**). The rest of the *L. aethiopica* genomes were trisomic at 1 to 6 chromosomes, including a group of 8 genomes that was trisomic for chromosome 1 (box 2 in **Figure 4**). The largest variability was found in *L. aethiopica* genome LEM3498, the *L. aethiopica* - *L. donovani* hybrid LEM3469 and its derived clones and the *L. aethiopica* - *L. tropica* hybrid L87 (box 3 in **Figure 4**). Altogether, our results demonstrate that *L. aethiopica* genomes are aneuploid, as shown for other *Leishmania* species. Moreover, note that we also observed non-integer values of somy for some chromosomes (**Figure 4**), suggesting the presence of mosaic aneuploidy (Negreira et al., 2022).

Local copy number variations were investigated using normalized haploid copy numbers (HCN) as estimated in non-overlapping 2kb windows. We identified a total of 379 genomic regions with decreased or amplified HCN across our panel of *L. aethiopica* genomes. The majority of these windows were only 2kb long (N = 266; 70%) and the mean (1.1 HCN) and median (0.79 HCN) differences in HCN across our panel were minor, suggesting that our sample of *L. aethiopica* displays little local copy number variation. Apart from this observation, most notable was a 46 kb telomeric genomic region on chromosome 29 in strain 1561-80 showing a 10-fold increase in HCN compared to the other *L. aethiopica* genomes. This region covers a total of 18 protein coding genes, including a ribonuclease inhibitor-like protein (LAEL147_000545200), an actin-like protein (LAEL147_000544400) and a putative inosine-adenosine-guanosine-nucleoside hydrolase (LAEL147_000545100) (**Supplementary Table 7**). Four other strains (L127, GEREcl7, 169-83 and 32-83) displayed intrachromosomal amplifications across genomic regions of 10 kb to 20 kb long, but these involved only 0.3 to 1.2 increase in HCN (**Supplementary Table 7**). Finally, small (<= 4kb) intrachromosomal amplifications were identified across the genomes of L100 and L100cl1 (**Supplementary Table 7**).

Within the *L. aethiopica* - *L. donovani* hybrid LEM3469 and its derived clones, we detected a 6kb genomic region in chromosome 29 showing a 2-fold increase in HCN and covering a Leucine Rich Repeat gene (LAEL147_000538100) (**Supplementary Table 8**). Within the *L. aethiopica* - *L. tropica* hybrid L87, we found a 130 kb genomic region in chromosome 35 with a 1.6 fold increase in HCN and covering a total of 46 protein coding genes (**Supplementary Table 9**). Within both hybrids, we detected a 14 kb - 16 kb genomic region in chromosome 30 showing a ~1-fold increase in HCN and covering protein coding genes such as a the putative ferric reductase gene (LAEL147_000563600) (**Supplementary Tables 8, 9**).

## 4 Discussion

We present the first comprehensive genome diversity study of *L. aethiopica* by analyzing high-resolution WGS data. This revealed insights into the genetic consequences of recombination in *Leishmania* at both the species- and population-level.

At the species level, we provide genomic evidence of hybridization involving *L. aethiopica* as one of the parental species and *L. tropica* (in case of the L86 hybrid strain) or *L. donovani* (in case of the LEM3469 hybrid strain) as the other parental species. Strain LEM3469 has already been described as a potential *L. aethiopica/L. donovani* hybrid based on microsatellite data and single-gene sequences (Odiwuor et al., 2011a). Strain L86 has been flagged as a potential hybrid, although it remained classified as *L. aethiopica* due to lack of molecular evidence (Odiwuor et al., 2011b). Here, our data provide genomic evidence that these two *Leishmania* strains are interspecific hybrids. Our finding of genome-wide heterozygosity that is only occasionally interrupted by patches of homozygosity suggest that these two hybrids are equivalent to F1 progeny that propagated mitotically since the initial hybridization event. Similar genomic descriptions of naturally circulating F1-like hybrids were advanced previously for *L. braziliensis* x *L. peruviana* in Peru (Van den Broeck et al., 2020) and for *L. braziliensis* x *L. guyanensis* in Costa Rica (Van den Broeck et al., 2022). Our results thus support a growing body of genomic evidence for extensive genetic exchange in protozoan parasites in the wild (Ramírez and Llewellyn, 2014; Rogers et al., 2014; Tihon et al., 2017; Van den Broeck et al., 2018; Inbar et al., 2019; Schwabl et al., 2019; Van den Broeck et al., 2020; Glans et al., 2021; Van den Broeck et al., 2022).

The *L. tropica/L aethiopica* (L86) hybrid strain was near diploid, being disomic at 27/36 chromosomes, suggesting balanced segregation of the chromosomes during hybridization. Trisomic chromosomes in L86 contained either one or two copies of the *L. tropica* parent, which may suggest that these trisomies arose after the hybridization event through multiplication of one of the parental chromosomes, possibly due to culturing *in vitro* (Dumetz et al., 2017). These observations seem unlikely to have arisen by a random parasexual process, and suggest that the L86 hybrid is the result of meiotic recombination. The *L. donovani/L aethiopica* (LEM3469) hybrid strain was near triploid, being trisomic at 21/36 chromosomes. Triploid hybrids have been routinely recovered from experimental matings in *Leishmania* (Inbar et al., 2019) and from clinical samples of cutaneous leishmaniasis patients (Van den Broeck et al., 2022), and are observed across a variety of organisms capable of sexual reproduction (Schultz and Dobzhansky, 1933; Hegarty and Hiscock, 2008; Stöck et al., 2010). Results from experimental crosses in *Leishmania* suggested that interspecific hybrids with close to 3n DNA content were likely due to an asymmetric meiosis between a parental 2n cell that failed to undergo meiosis and 1n gamete from the other parent (Inbar et al., 2019), similar to what has been suggested for *T. brucei* (Gibson and Stevens, 1999). One observation that requires further investigation is that triploid hybrids may occur more frequently when the two parental species are genetically divergent (in the case of *L. donovani/L aethiopica*, *L. guyanensis/L. braziliensis* (Van den Broeck et al., 2022) and *L. infantum/L. major* (Inbar et al., 2019) hybrids), while diploid hybrids seem to occur when the parental species are more closely related (in the case of *L. tropica/L. aethiopica* and *L. braziliensis/L. peruviana* (Van den Broeck et al., 2020) hybrids).

At the population level, our analyses of sequence variation confirmed previous observations that the *L. aethiopica* species is genetically diverse, despite its restricted geographic distribution (Schönian et al., 2000; Pratlong et al., 2009; Krayter et al., 2015). Indeed, we detected on average 91 SNPs per 10kb in *L. aethiopica*, which is comparable to the genetically diverse *L. braziliensis* (106 SNPs per 10kb) but twice the number observed in the demographically bottlenecked *L. peruviana* (41 SNPs per 10kb) (Van den Broeck et al., 2020). By comparison, the human genome contains ~8 SNPs per 10kb (Zhao et al., 2003). Also, the total number of SNPs (284,776) across our set of 20 *L. aethiopica* genomes from Ethiopia is of the same magnitude as the number of SNPs (395,624) observed in a set of 151 globally sampled genomes of the *L. donovani* species complex (Franssen et al., 2020). In contrast, we detected little variation in local copy numbers: the majority of genomes contained only up to 20 genomic regions with increased/decreased read depths. Hence, adaptation in our sample of *L. aethiopica* genomes may depend on sequence variation rather than gene dosage. Future studies involving direct sequencing of biopsy samples (Domagalska et al., 2019) should allow deciphering the relative contribution of different genomic variants in clinical outcome or treatment failure.

Population genomic analyses revealed that the *L. aethiopica* species consists of both asexually evolving strains (as indicated by the existence of near-identical genomes) and groups of parasites that show signatures of recombination (as indicated by linkage decay). In addition, parasite populations were genetically structured, suggesting that *L. aethiopica* consists of divergent populations, although lack of geographic/ecological data precluded us from studying its evolutionary history in greater detail. A remarkable observation was the extensive LOH in some *L. aethiopica* strains. Such LOH likely arose from gene conversion/mitotic recombination and indicates that *L. aethiopica* strains may evolve asexually over long time periods. This is exemplified by two near-identical genomes (678-82 and LEM2358cl3) that were sampled nine years from each other. Interestingly, we observed four additional LOH regions in LEM2358cl3 (sampled in 1991) impacting an additional 50 kb of the nuclear genome compared to 678-82 (sampled in 1982), suggesting that nine years of clonal evolution has resulted in LOH across 50kb of the genome.

Extensive LOH regions have been described previously for obligate asexual eukaryotes, such as the water flea *Daphnia pulex* (Tucker et al., 2013) and the protozoan parasite *Trypanosoma brucei gambiense* (Weir et al., 2016; Geerts et al., 2022). In *T. b. gambiense*, most LOH regions are ancestral and thus present in all strains, or at least within sets of strains that share a common ancestry (Weir et al., 2016). In contrast, we observed large differences in the number and length of LOH regions between *L. aethiopica* strains, irrespective of their ancestral relationships. For instance, LOH regions were absent in the L127 strain while a total of 154 LOH regions were found in the L100 strain, impacting at least 82% of its nuclear genome. The L100 strain (also known as LEM144) was isolated in 1972 and has - to the best of our knowledge - only been cultured *in vitro* for about 30 passages before sequencing. Hence, the extensive loss of heterozygosity cannot be explained by maintenance *in vitro*, and is probably due to long-term mitotic recombination in the wild, as described for the asexually evolving *T. b. gambiense* (Weir et al., 2016). Alternatively, the genome-wide loss-of-heterozygosity in L100 may be explained by genome-wide DNA haploidization, possibly due to the production of haploid gametes as seen in trypanosomes (Peacock et al., 2021), followed by whole-genome diploidization (as our analyses demonstrated that L100 is diploid). However, there is currently no evidence in literature for the occurrence of whole-genome haploidization/diploidization in *Leishmania* parasites.

In conclusion, our study has shown that *L. aethiopica* is genetically diverse and divided into structured populations, suggesting that strains may be ecologically/geographically confined. While linkage decay suggests the occurrence of genetic exchange, the discovery of extensive loss of heterozygosity also suggests that some *L. aethiopica* strains may evolve asexually for long periods of time. Hence, *L. aethiopica* presents an ideal model to understand the impact of parasite population structure and hybridization on genome evolution in protozoan parasites. Our preliminary observations should thus be investigated in further detail using larger sets of samples for which detailed eco-epidemiological data are available, preferably using direct-sequencing approaches that would also allow understanding the genomic basis of clinical and treatment outcome.

## Data availability statement

Genomic sequence reads of all sequenced genomes are available on the European Nucleotide Archive (https://www.ncbi.nlm.nih.gov/bioproject/PRJNA924694) under accession number PRJNA924694.

## Author contributions

J-CD and MD conceived the original idea. IM performed the lab experiments. FB and SH supervised the work of AH. AH, SH, and FB performed the computational analyses and wrote the manuscript. All authors discussed the results. All authors contributed to the article and approved the submitted version.
