# Supplementary material for: Genome diversity of Leishmania aethiopica
Source: Front Cell Infect Microbiol. 2023 Apr 20;13:1147998. doi: 10.3389/fcimb.2023.1147998 (PMC10157169; doi:10.3389/fcimb.2023.1147998)
Supplement: Supplementary file 1 [file DataSheet_1.zip › SuppFigures.pdf]

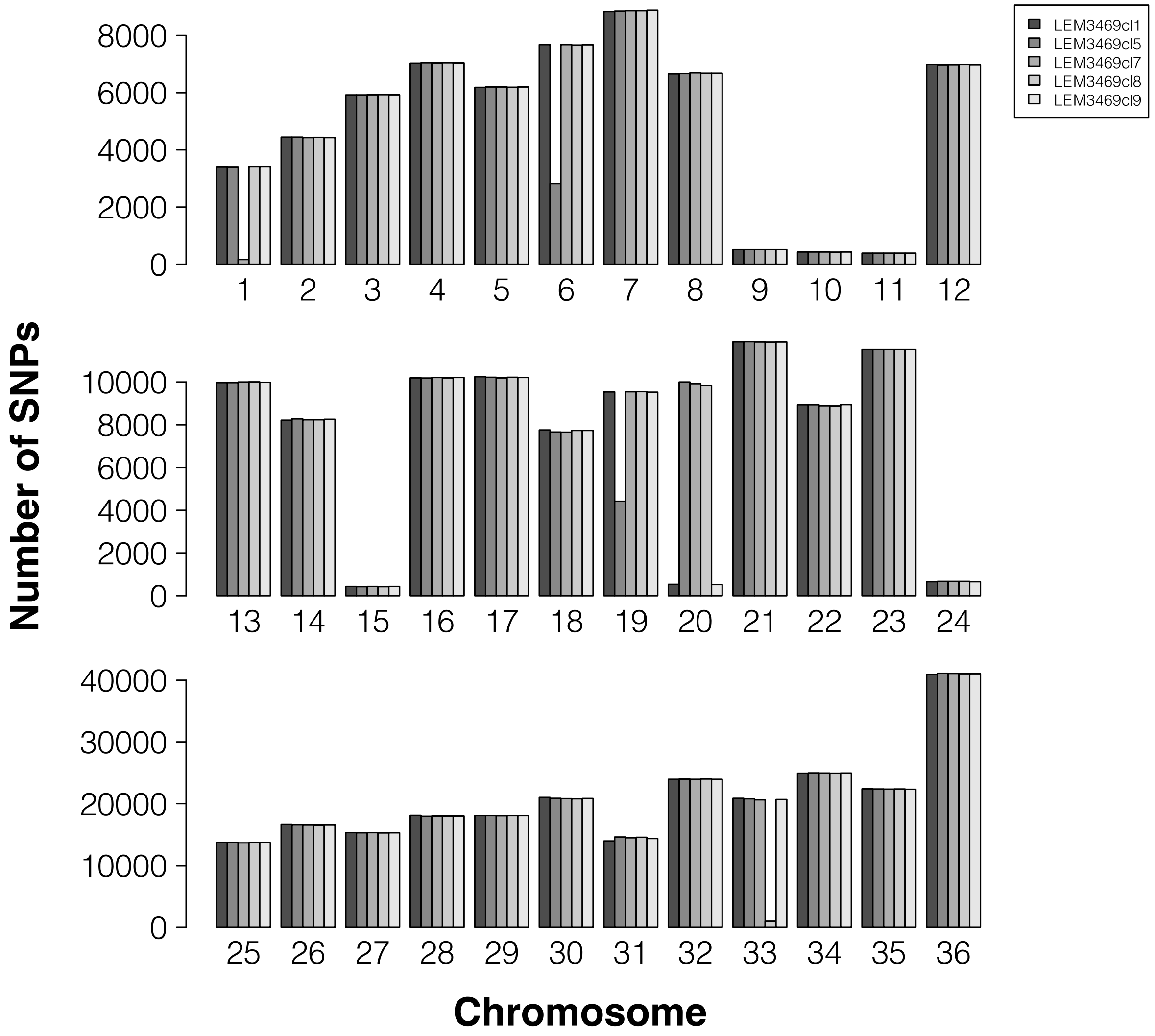

Supplementary Figure 1. Number of SNPs for each of the 36 chromosomes in LEM3469 and its five derived clones.

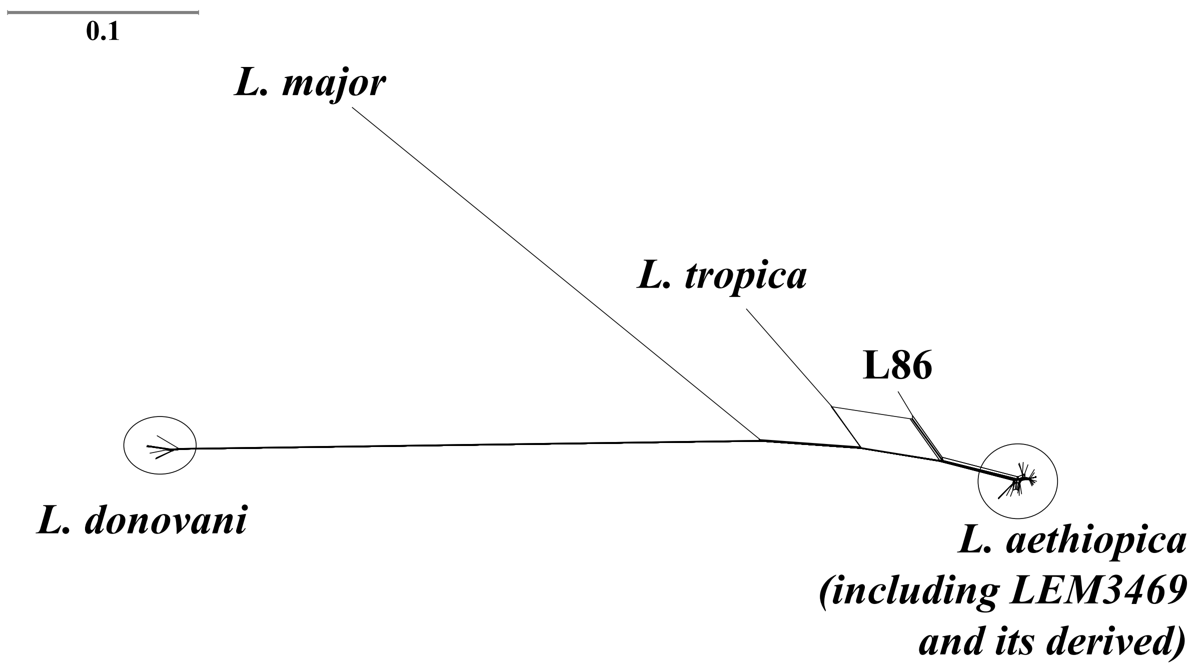

Supplementary Figure 2. Phylogenetic network based on SNPs of chromosome 15 that were called across 36 genomes of *L. aethiopica*, the *L. donovani* species complex, *L. major* and *L. tropica*.

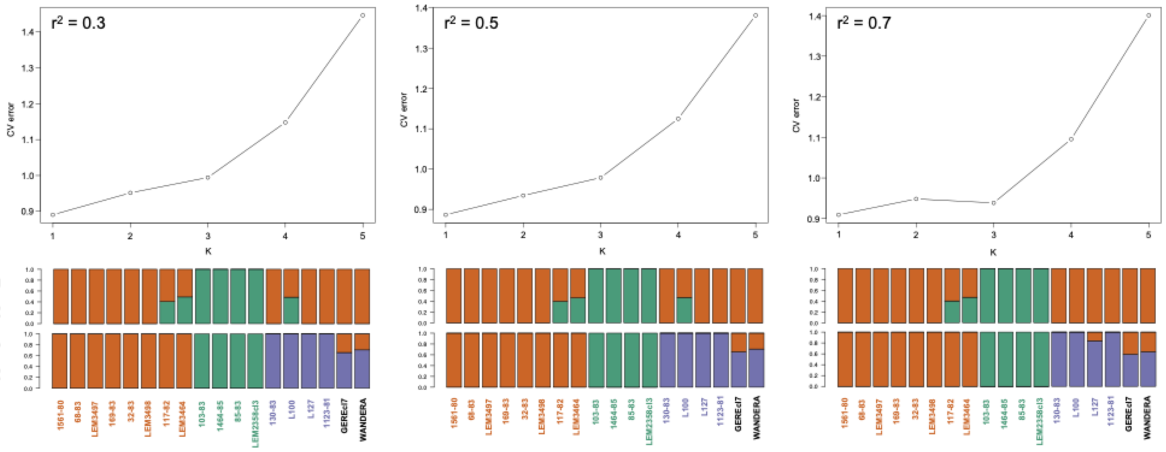

Supplementary Figure 3. Model-based ancestry estimation of *L. aethiopica*, as inferred by ADMIXTURE, for different SNP-pruning thresholds (see methods). Upper panels depict the 5-fold cross validation plots for K = 1-5. Lower panels represent barplots of the ancestral components inferred by ADMIXTURE for K = 2 and K = 3. (left) SNP pruning at r^2=0.3 retaining 47,244 SNPs. (middle) SNP pruning at r^2=0.5 retaining 85,725 SNPs. (right) SNP pruning at r^2=0.7 retaining 112,241 SNPs.

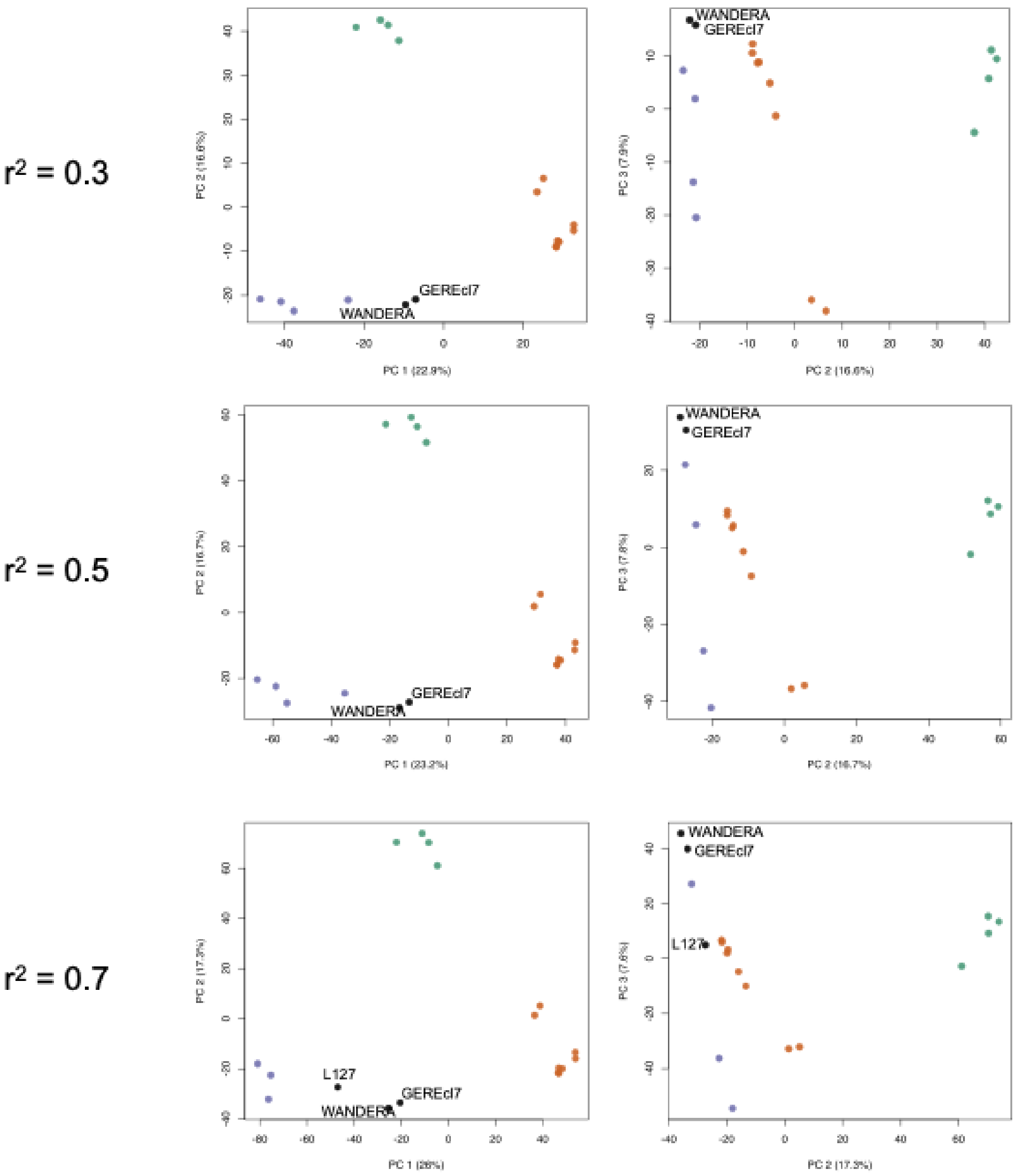

Supplementary Figure 4. Principal component analysis for the different SNP-pruning thresholds (see methods). (upper) SNP pruning at r^2=0.3 retaining 47,244 SNPs. (middle) SNP pruning at r^2=0.5 retaining 85,725 SNPs. (lower) SNP pruning at r^2=0.7 retaining 112,241 SNPs. Colors represent the population assignment as inferred by ADMIXTURE (Supplementary Figure 3).

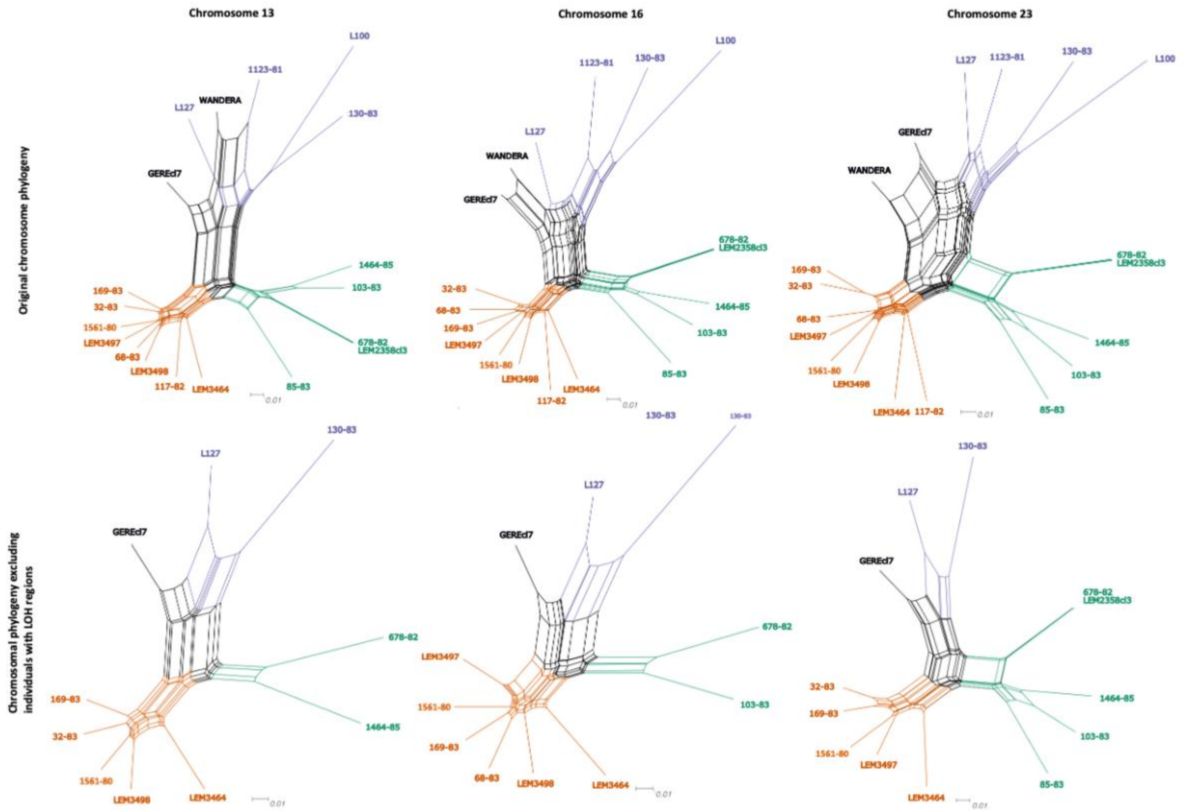

Supplementary Figure 5. Phylogenetic network based on uncorrected p-distances for chromosome 13 (left), 16 (middle) and 23 (right) with (lower) and without (upper) excluding individuals containing LOH regions. Coloured branches and tip labels correspond to the inferred populations by ADMIXTURE at K=3 (Figure 3A).
